# Functionally-Coupled Ion Channels Begin Co-assembling at the Start of Their Synthesis

**DOI:** 10.1101/2025.04.08.647703

**Published:** 2026-01-12

**Authors:** Roya Pournejati, Jessica M. Huang, Michael Ma, Claudia M. Moreno, Oscar Vivas

## Abstract

Calcium binding to BK channels lowers BK activation threshold, substantiating functional coupling with calcium-permeable channels. This coupling requires close proximity between different channel types, and the formation of BK–Ca _V_ 1.3 hetero-clusters at nanometer distances exemplifies this unique organization. To investigate the structural basis of this interaction, we tested the hypothesis that BK and Ca _V_ 1.3 channels assemble before their insertion into the plasma membrane. Our approach incorporated four strategies: (1) detecting interactions between BK and Ca _V_ 1.3 proteins inside the cell, (2) identifying membrane compartments where intracellular hetero-clusters reside, (3) measuring the proximity of their mRNAs, and (4) assessing protein interactions at the plasma membrane during early translation. These analyses revealed that a subset of BK and Ca _V_ 1.3 transcripts are spatially close in micro-translational complexes, and their newly synthesized proteins associate within the endoplasmic reticulum (ER) and Golgi. Comparisons with other proteins, transcripts, and randomized localization models support the conclusion that BK and Ca _V_ 1.3 hetero-clusters form before their insertion at the plasma membrane.

**Significance statement:**

This work examines the proximity between BK and Ca _V_ 1.3 molecules at the level of their mRNAs and newly synthesized proteins to reveal that these channels interact early in their biogenesis. Two cell models were used: a heterologous expression system to investigate the steps of protein trafficking and a pancreatic beta cell line to study the localization of endogenous channel mRNAs. Our findings show that BK and Ca _V_ 1.3 channels begin assembling intracellularly before reaching the plasma membrane, revealing new aspects of their spatial organization. This intracellular assembly suggests a coordinated process that contributes to functional coupling.

## Full Text Availability

The license terms selected by the author(s) for this preprint version do not permit archiving in PMC. The full text is available from the preprint server.

