# Supplementary material for: Functionally-Coupled Ion Channels Begin Co-assembling at the Start of Their Synthesis
Source: bioRxiv. 2025 Nov 4:2025.04.08.647703. Preprint. [Version 3] doi: 10.1101/2025.04.08.647703 (PMC12637664; doi:10.1101/2025.04.08.647703)
Supplement: Supplementary file 1 [file NIHPP2025.04.08.647703v3-supplement-1.pdf]

bioRxiv preprint doi: https://doi.org/10.1101/2025.04.08.647703; this version posted November 4, 2025. The copyright holder for this preprint (which was not certified by peer review) is the author/funder, who has granted bioRxiv a license to display the preprint in perpetuity. It is made available under aCC-BY 4.0 International license.

Supplementary figures

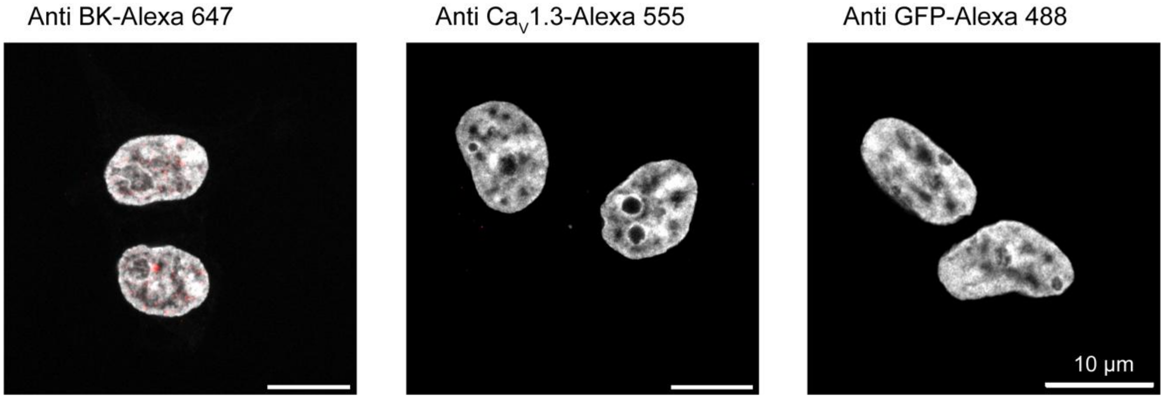

Figure 2-Supplementary 1. Validation of antibodies against BK, Ca_V1.3 and GFP. Representative confocal images of tsA-201 cells immuno-tested for BK channels (left), Ca_V1.3 channels (middle), and GFP proteins (right). Cells were not transfected. Nuclei were stained with DAPI and pseudo colored in gray. Scale bar is 10 µm for all images.

bioRxiv preprint doi: https://doi.org/10.1101/2025.04.08.647703; this version posted November 4, 2025. The copyright holder for this preprint (which was not certified by peer review) is the author/funder, who has granted bioRxiv a license to display the preprint in perpetuity. It is made available under aCC-BY 4.0 International license.

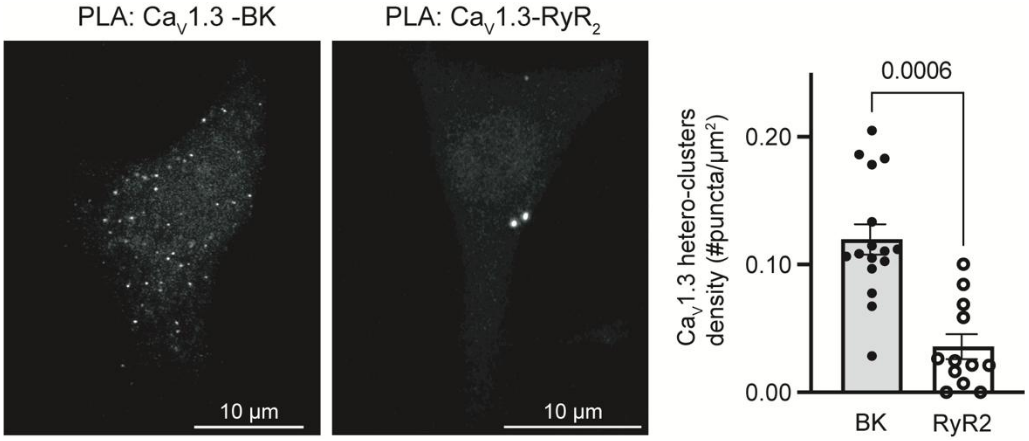

Figure 4-Supplementary 1. $Ca_V1.3$ channels do not exhibit proximity interactions with ryanodine receptor type 2 (RyR_2). A. Representative images of proximity ligation assay (PLA) experiments in INS-1 cells labeling against $Ca_V1.3$ and BK channels (left) or $Ca_V1.3$ and RyR_2 (right). Comparison of PLA puncta density for the two conditions. Statistical significance was assessed using an unpaired t-test.

bioRxiv preprint doi: https://doi.org/10.1101/2025.04.08.647703; this version posted November 4, 2025. The copyright holder for this preprint (which was not certified by peer review) is the author/funder, who has granted bioRxiv a license to display the preprint in perpetuity. It is made available under aCC-BY 4.0 International license.

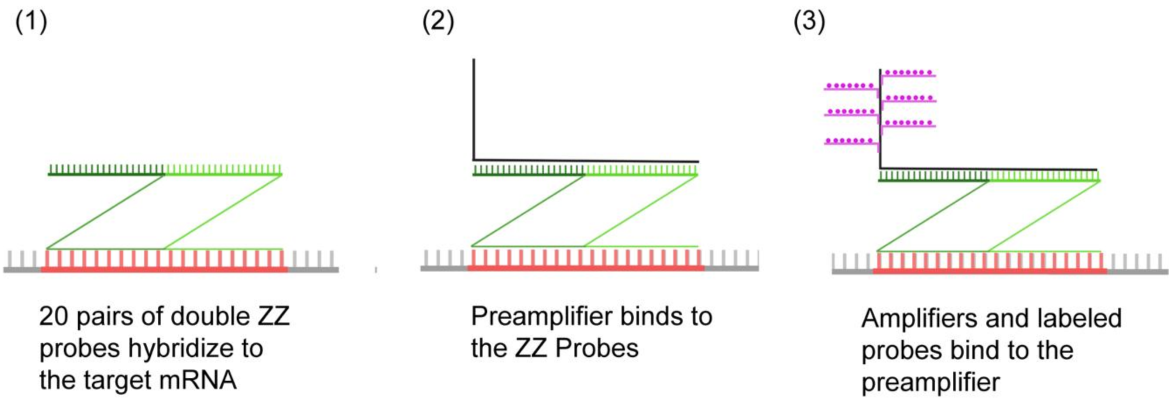

Figure 5-Supplementary 1. Illustration of RNA scope methodology. Steps to detect an mRNA sequence (red zipper) consisting of (1) hybridization, (2) pre-amplification, (3) and labeling. Double ZZ probes are shown in green. Dye labeling the mRNA is shown in magenta.

bioRxiv preprint doi: https://doi.org/10.1101/2025.04.08.647703; this version posted November 4, 2025. The copyright holder for this preprint (which was not certified by peer review) is the author/funder, who has granted bioRxiv a license to display the preprint in perpetuity. It is made available under aCC-BY 4.0 International license.

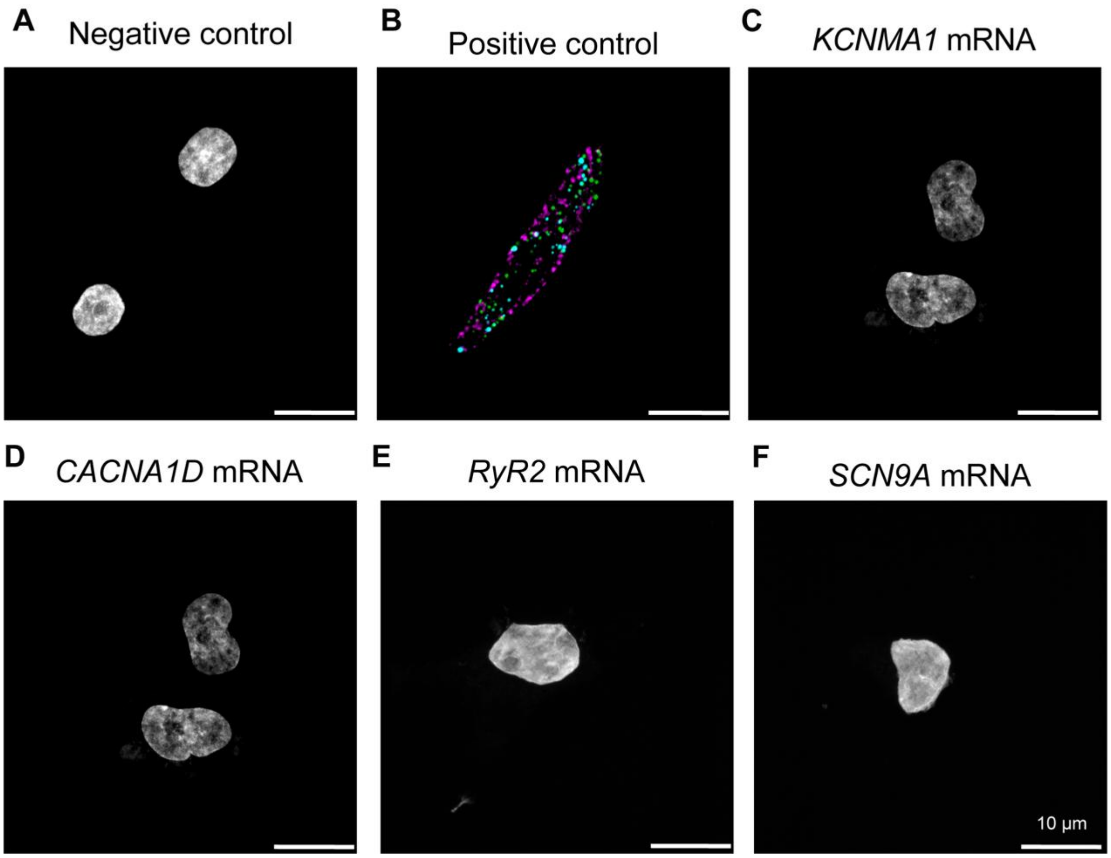

Figure 5-Supplementary 2. RNA probe validation. A. Representative images of INS-1 cells subjected to mRNA probes against a bacterial gene (DapB from *Bacillus subtilis*). **B.** Representative images of INS-1 cells subjected to mRNA probes against three constitutive mammalian genes (ubiquitin C, cyclophilin B, and a polymerase II subunit). **C - F** show tsA-201 cells that were naive to exogenous DNA but treated with mRNA probes against the genes for (**C**) BK channels, (**D**) CaV1.3 channels, (**E**) Ryanodine Receptors type 2 (RyR2), and (**F**) NaV1.7 channels. Scale bar is 10 μm.

bioRxiv preprint doi: https://doi.org/10.1101/2025.04.08.647703; this version posted November 4, 2025. The copyright holder for this preprint (which was not certified by peer review) is the author/funder, who has granted bioRxiv a license to display the preprint in perpetuity. It is made available under aCC-BY 4.0 International license.

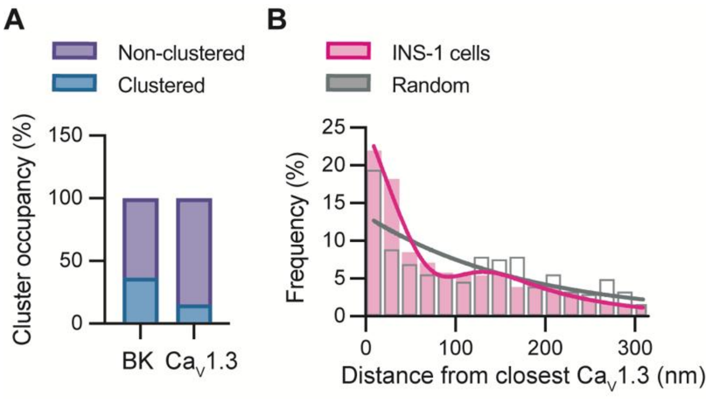

Figure 8-Supplementary 1. Organization of BK-CaV1.3 hetero-clusters in INS-1 cells. A. Proportion of channels in clusters or outside clusters for BK and CaV1.3. Clustered channels were defined as being at a distance of 200 nm or less from the other channel type. Channels found at 200 nm or more were considered non-clustered. Data from 7 cells. **B.** Distribution of the nearest neighbor distance from BK channels to CaV1.3 channels in INS-1 cells (magenta, n = 1037 BK channels, from N = 5 cells). Distribution of the nearest neighbor distance from BK channels to randomized channels (gray, n = 363 BK channels). The shift in the distribution from INS-1 cells to randomized images suggests a preferential localization of endogenous BK-CaV1.3 hetero-clusters.
